# Measurement of various intensities of physical activities and categorization of “Locomotive” and “Household” activities provide a subject-specific detailed assessment

**DOI:** 10.1038/s41598-021-99392-9

**Published:** 2021-11-11

**Authors:** Ryuichiro Inaba, Satoshi Yamakawa, Takashi Kanamoto, Sho Ukimoto, Seira Sato, Issei Ogasawara, Shoji Konda, Teruki Yokoyama, Yuko Ueda, Takashi Onuki, Ken Nakata

**Affiliations:** 1 Department of Medicine for Sports and Performing Arts, Graduate School of Medicine, Osaka University, Suita, Osaka 565-0871 Japan; 2 Department of Sports Medical Biomechanics, Graduate School of Medicine, Osaka University, Suita, Osaka 565-0871 Japan; 3 Department of Biomechanics and Motor Control, Graduate School of Medicine, Osaka University, Toyonaka, Osaka 560-0043 Japan; 4 Department of Sports Medical Science, Graduate School of Medicine, Osaka University, Suita, Osaka 565-0871 Japan

**Keywords:** Health care, Public health, Quality of life

## Abstract

This study aimed to compare the physical activity (PA) measured by a wearable sensor device (WSD) and the step count measurement, and to investigate the association between PAs and lifestyle. Data of 301 participants were collected from March 2019 to March 2021. Step counts, sedentary behavior, performance time of light/moderate/vigorous PA, METs × hour of “Locomotive” and “Household” categorized activities, and energy expenditure (EE) were measured by the WSD, respectively. Furthermore, the participants were classified into student, standing worker, and sitting worker groups. Data were analyzed using the Steel–Dwass and Pearson correlation coefficient tests. The correlation between the performance time of each PA and step count was weak, except for moderate PA. “Household” EE and step count also had a weak correlation. In the comparison of lifestyle, there was a significant difference in the mean performance time of each type of PA between the groups. Additionally, the standing worker and sitting worker groups had a significant difference in METs × hour of "Household" activities, indicating that the difference between the occupations is reflected in “Household” activities. The WSD measurement can be used to evaluate detailed individual PA, whereas the step count measurement showed weakness in the PA estimation.

## Introduction

Physical inactivity is a common health issue for people worldwide^1^. It is well known as a risk factor for developing non-communicable diseases (NCDs), such as coronary heart disease, type 2 diabetes, and breast and colon cancers^2^. Physical inactivity is the fourth leading cause of death worldwide^3^, and NCDs accounted for more than 70% of all deaths in 2017^4^. Resolving the issue of physical inactivity reduces the risk of developing NCDs by 6–10% and significantly contributes to increasing life expectancy^2^. Many countries and institutions have developed guidelines for the recommendation of daily exercise for the solution of physical inactivity^5–9^. For example, the Ministry of Health, Labor and Welfare of Japan recommends performing physical activity (PA) with the same intensity as moderate walking or higher intensity PA for 60 min daily for people aged 18–64 years. It is approximately the same as 8000 to 10 000 steps/day, including low-intensity level walking^6^. However, it is necessary to quantify the amount of PA to better understand the activities that have been carried out in person.

Many investigators have attempted to quantify PA, and several methodologies have been developed. The International Physical Activity Questionnaire and Global Physical Activity Questionnaire have been developed as subjective questionnaire methods and have become common in general health investigations^10,11^. In these methodologies, it is difficult to analyze detailed PA situations, such as the intensity of the activities and its performance time because of the participant's self-assessment^12^. However, investigators focused on walking, which is one of the major PAs in human daily activities, and evaluated PA using a pedometer^13^. Although the step counts could roughly evaluate all PAs on 1 day, it was difficult to measure the intensity of the PA and its performance time. First, the definition of PA is, "any bodily movement produced by skeletal muscles that require energy expenditure"^14^. This definition means that the PA includes not only sports activities but also activities of daily living, such as housework, commuting, shopping, gardening, and leisure activities. Thus, quantifying the intensity of PA is necessary to understand the PA situation and assess the effect of the situation on one’s health condition. In fact, the World Health Organization (WHO) developed guidelines based on PA intensity^7^. The intensity of PA is described by METs. The baseline of METs is set at no activity state with supine posture, and it is described as "1” and/or “sedentary behavior (SB)." Then, based on 1 METs, it is classified into three states: light physical activity (LPA: 1.5–2.9 METs), moderate physical activity (MPA: 3.0–5.9 METs), and vigorous physical activity (VPA: > 6.0 METs). In addition, MPA and VPA are often described together as moderate to vigorous physical activity (MVPA)^15^. In 2020, WHO revised PA guidelines which has previously recommended, recommending that adults aged 18–64 should do > 150 min of MPA or > 75 min VPA per week^7^.

In recent research, wearable sensor devices (WSDs) equipped with accelerometers have been featured^13,16,17^. These devices can measure the PA intensity and its performance time by measuring three-dimensional body acceleration. Some reports have indicated the usability of the device, and it has also been used in large-scale studies^13,18–20^. The WSD has been functionally improved daily, and some models provide interesting functions. For example, a WSD that can categorize PA into "Locomotive" and "Household" activities has emerged^21,22^. Basically, "Locomotive" activities are recognized as activities with steps, such as walking and running, and whereas "Household" activities are recognized as activities without steps, such as doing laundry and dishwashing. These categories of PA are useful for characterizing an individual’s lifestyle with a quantitative measurement of each type of PA. However, previous studies mainly focused on sports activities categorized as "Locomotive" activities, even though the "Household" activities are also important for PA evaluation in all daily individual activities. Thus, more detailed investigations focused on both "Locomotive" and "Household" activities are required for PA assessment. The WSD is expected to develop application tools that continuously measure PA in several fields. Many reports have compared PA measured using the WSD to a questionnaire^23,24^. However, there are few reports comparing the WSD-measured PA and step counts. Additionally, in people’s recently diversified lifestyles, more detailed PA data are required, and it can be expected that characterizing lifestyles from the obtained PA data will lead to more active PA implementation. Therefore, the objectives of the present study were to compare the PA measured by a WSD and the step count data, and to investigate the association between PAs and lifestyle.

## Results

**Figure 1:**
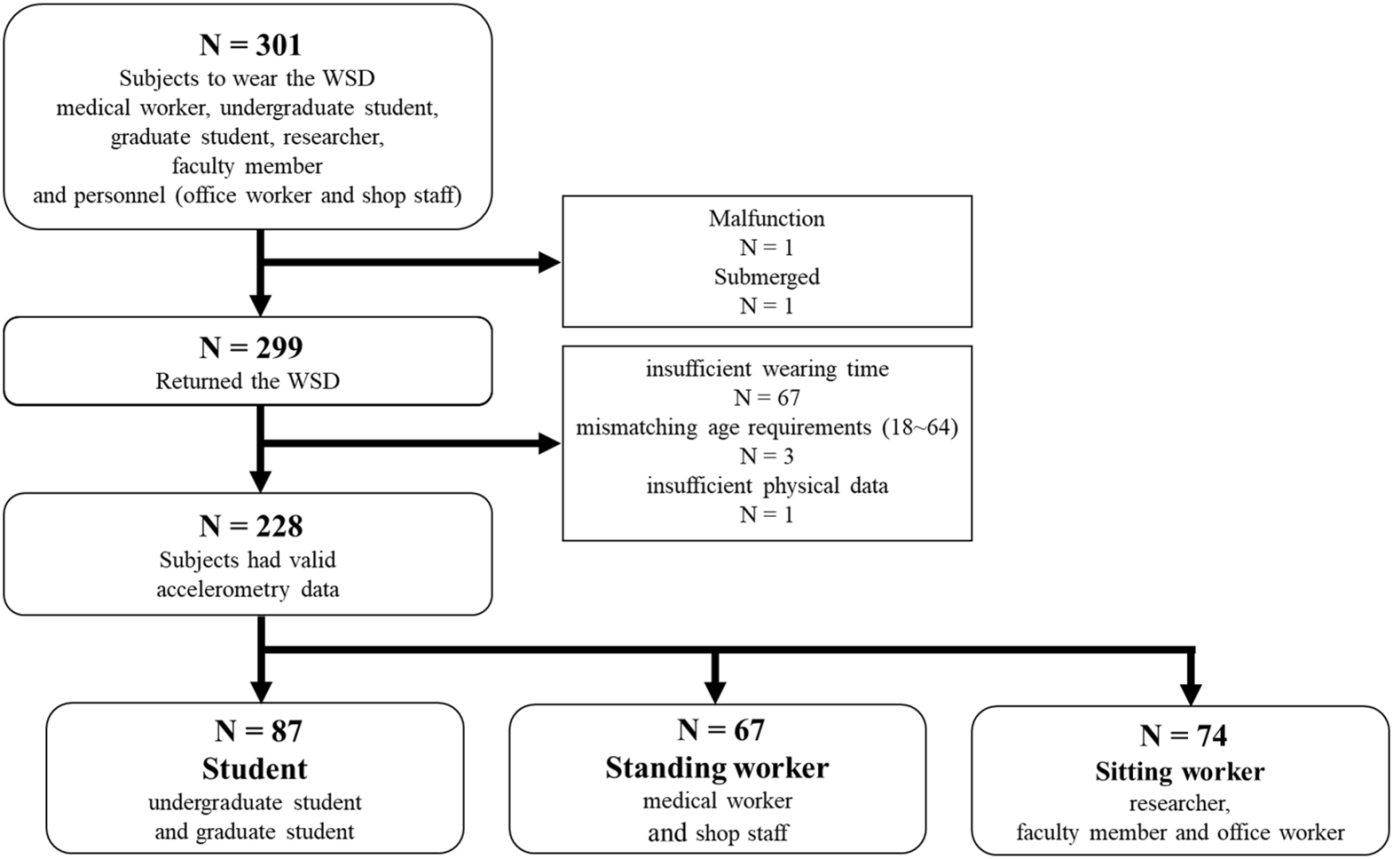
Flow chart showing the selection process of participants in the present study. *WSD* wearable sensor device.

Seventy-three of 301 participants were excluded from the study because of mismatching age requirements (n = 3), insufficient wearing time (n = 67), insufficient physical data (n = 1), malfunction of the device (n = 1), and submerged device (n = 1) (Fig. 1). The included participants were 228 with valid accelerometer data (69 men; age, 36.3 ± 14.3 years; body mass index [BMI] 21.1 ± 2.6 kg/m^2^; Table 1).

**Table 1:** Participant characteristic.

	All	Student	Standing worker	Sitting worker
N (men)	228 (69)	87 (43)	67 (10)	74 (16)
Age (y)	36.3 ± 14.3	20.4 ± 3.2^b,c^	46.4 ± 7.6^a^	45.8 ± 9.2^a^
Height (cm)	162.6 ± 7.8	164.9 ± 8.4^b^	160.1 ± 7.5^a^	162.1 ± 6.7
Weight (kg)	56.0 ± 9.4	56.8 ± 9.0	54.1 ± 9.3	56.9 ± 9.8
BMI (kg/m^2^)	21.1 ± 2.6	20.8 ± 2.1	21.0 ± 2.6	21.6 ± 3.0
BMI categories				
Nomal (< 25.0 kg/m^2^)	213	84	63	66
Obese (≥ 25.0 kg/m^2^)	15	3	4	8

*BMI* body mass index; *vs.* versus.

Values are expressed as the mean ± standard deviation.

a: *p* < 0.05 vs. Student group.

b: *p* < 0.05 vs. Standing worker group.

c: *p* < 0.05 vs. Sitting worker group.

**Figure 2:**
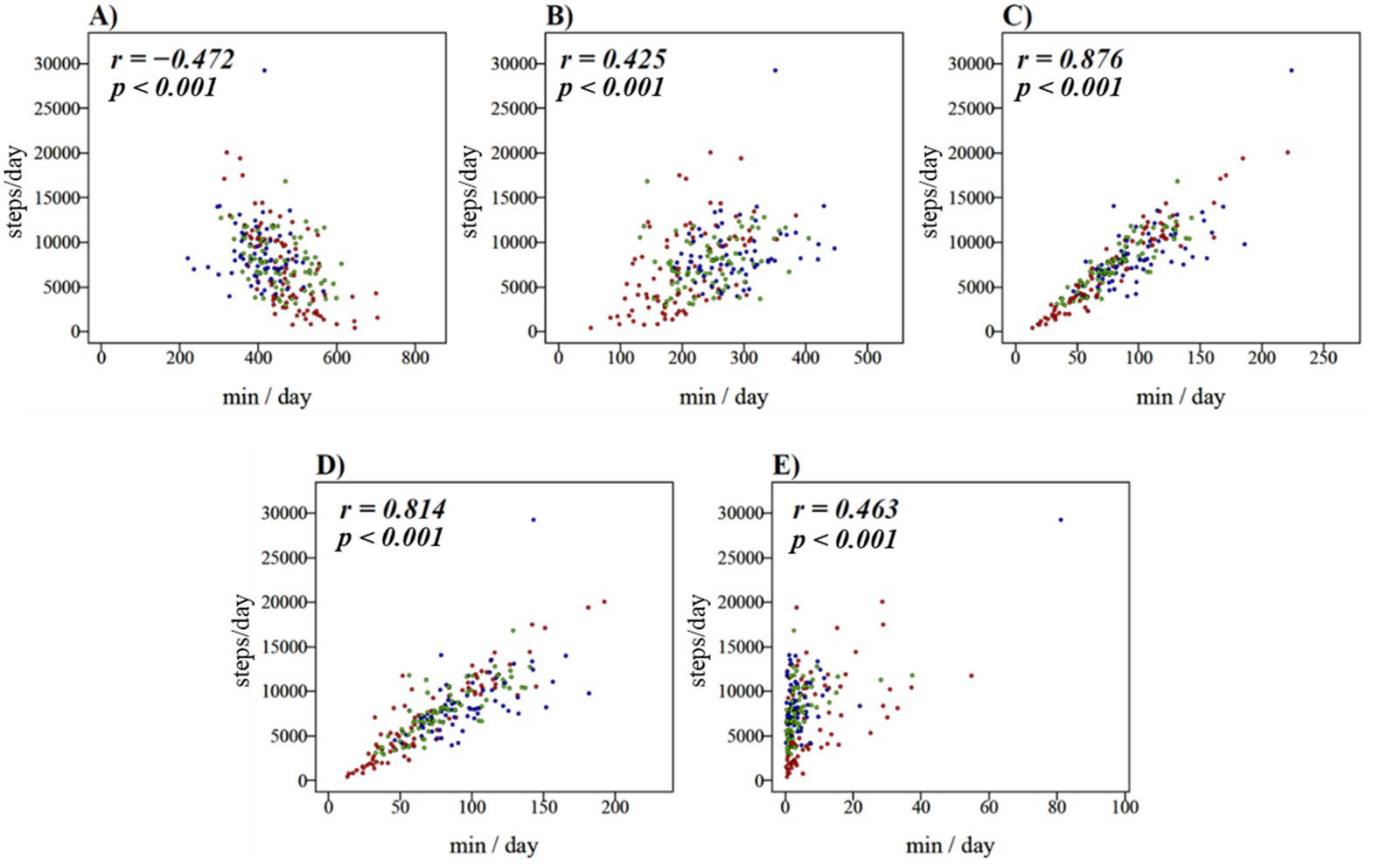
Scatterplots depicting the relationship between each type of physical activity and step counts of the total participants. **(A)** Sedentary behavior and step counts, (**B)** light physical activity and step counts, (**C)** moderate to vigorous physical activity and step counts, (**D)** moderate physical activity and step counts, (**E)** vigorous physical activity and step counts. : Student group; : Standing worker group; : Sitting worker group.

**Figure 3:**
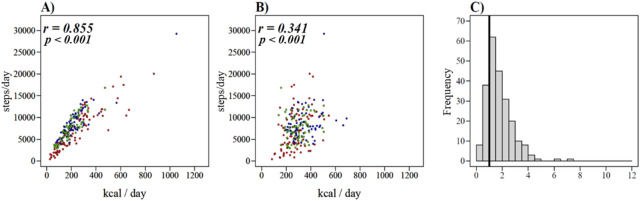
Scatterplots depicting the relationship between energy expenditure (EE) and step counts of the total sample and ratio of “Locomotive” and “Household” EE. (**A)** “Locomotive” EE and step counts, (**B)** “Household” EE and step counts, (**C)** histogram of the ratio of “Household” EE to “Locomotive” EE. (The vertical line (*x* = 1) means that the EE of “Locomotive” activities and “Household” activities in a day are equal). : Student group; : Standing worker group; : Sitting worker group.

In analysis of the correlation between the step counts and performance time of each type of PA, the SB showed a negative correlation (Fig. 2A), and the LPA, MVPA, MPA, and VPA showed a positive correlation (Fig. 2B–E). The correlation between step counts and MPA and MVPA was strong (*r* = 0.876 and *r* = 0.814, respectively), whereas the correlation between step counts and SB, LPA, and VPA was moderate (*r* =  − 0.472, *r* = 0.425, and *r* = 0.463, respectively). In addition, a strong correlation was found between the step counts and “Locomotive” EE (*r* = 0.855) (Fig. 3A), whereas a weak correlation was found between the step counts and “Household” EE (*r* = 0.341) (Fig. 3B). Overall, 79.8% of participants showed a higher EE in “Household” activities than that of “Locomotive” activities (Fig. 3C).

**Figure 4:**
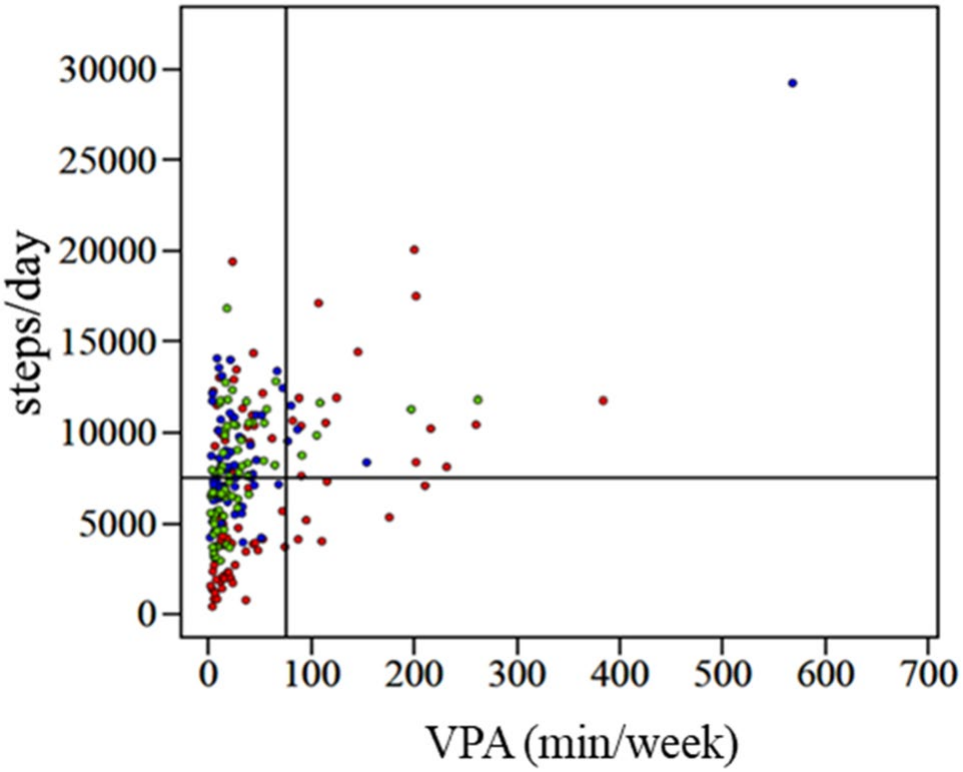
Scatterplots depicting the relationship between vigorous physical activity (VPA) (per week) and step counts of all participants. The vertical line indicates the VPA guidelines (≥ 75 min/week) recommended by the World Health Organization, and the horizontal line indicates the step count recommendation for older women (≥ 7500 steps/day), as reported by Lee et al.^25^. : Student group; : Standing worker group; : Sitting worker group. Upper right: first quadrant; upper left: second quadrant; lower left: third quadrant; lower right: fourth quadrant.

All participants were divided into four groups based on the target line for the step counts and VPA performance time from the WHO recommendations^7^ (performing the VPA for 75 min a week) and Lee et al.’s study^25^ (performing 7500 steps a day). The first quadrant included 25 participants (11.0%), second quadrant included 91 participants (39.9%), third quadrant included 106 participants (46.5%), and fourth quadrant included six participants (2.6%) (Fig. 4).

**Figure 5:**
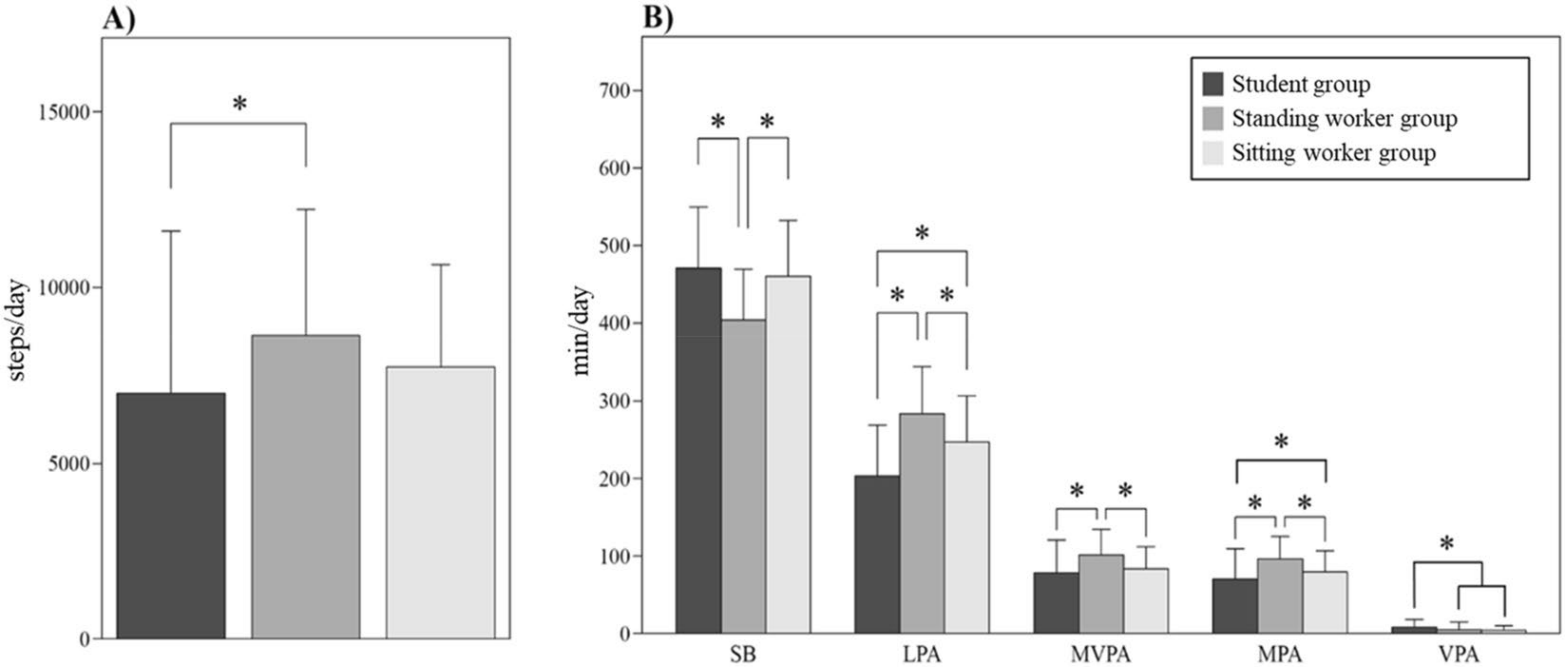
Step counts **(A)** and mean performance time of sedentary behavior (SB) and each type of physical activity **(B)** in the student, standing worker and sitting worker groups. *LPA* light physical activity; *MVPA* moderate to vigorous physical activity; *MPA* moderate physical activity; *VPA* vigorous physical activity. *p < 0.05.

**Figure 6:**
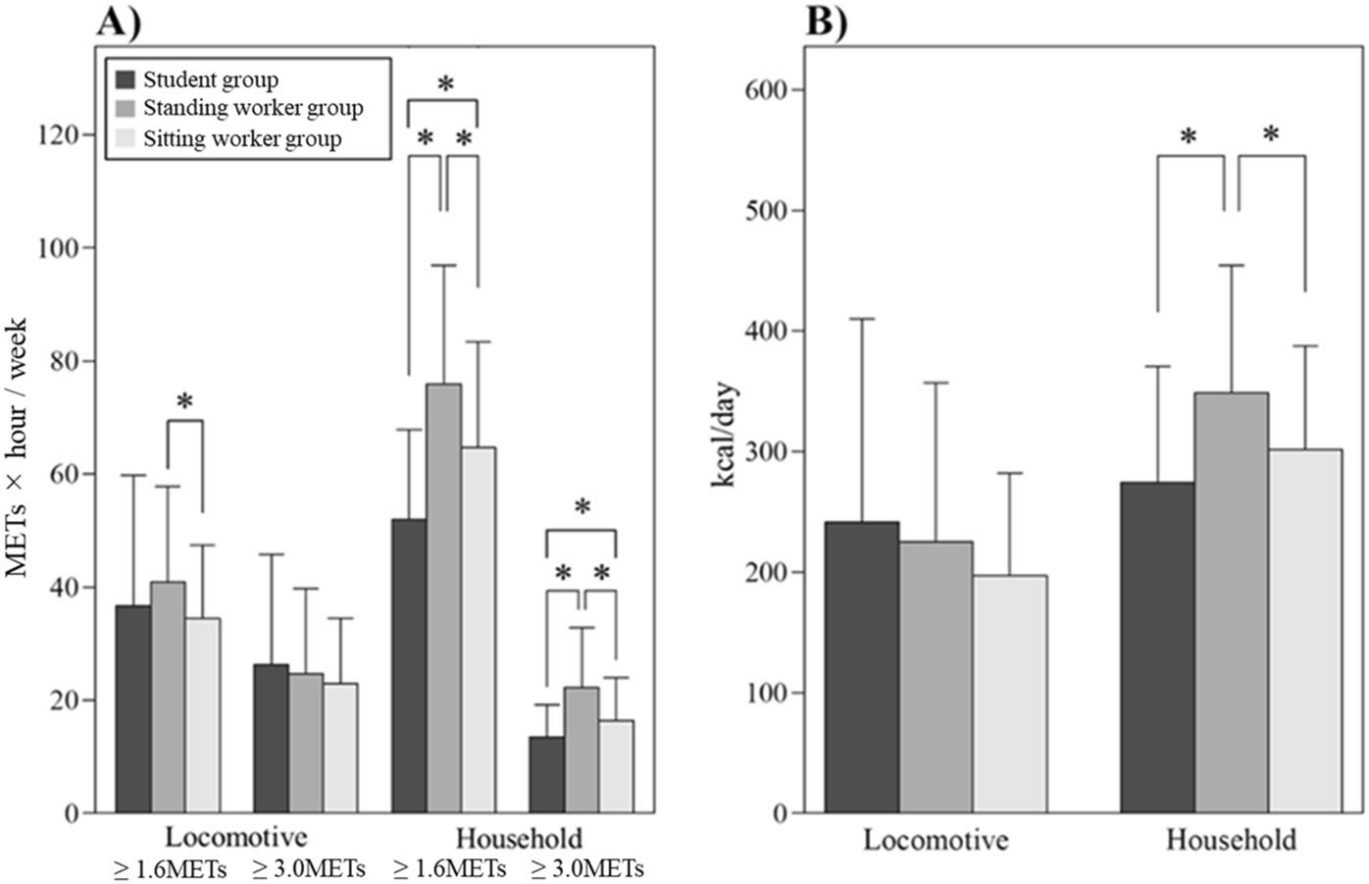
Magnitude of exercise (METs × hour) **(A)** and energy expenditure **(B)** of “Locomotive” and “Household” activities in the student, standing worker, and sitting worker groups. *p < 0.05.

Regarding lifestyle, the age of the student group was significantly lower than that of the other groups, and the height of the standing worker group was significantly lower than that of the student group. There were no significant differences in body weight and BMI between the groups (Table 1). The student group had a significantly shorter wearing time than the standing worker and sitting worker groups (753.5 ± 72.5 min/day, 789.9 ± 79.4 min/day, and 791.7 ± 92.1 min/day, respectively; *p* = 0.014 and *p* = 0.031, respectively). Regarding step counts, the student group was significantly smaller than the standing worker group (*p* = 0.015) (Fig. 5A). Regarding the PA performance time, the student group had a significantly longer SB performance time (*p* < 0.001) and significantly shorter LPA, MPA, and MVPA performance times than the standing worker group (*p* < 0.001, *p* < 0.001, and *p* < 0.001, respectively), and the student group had significantly shorter LPA and MPA performance times than the sitting worker group (*p* < 0.001 and *p* = 0.036, respectively). The sitting worker group had a significantly longer SB performance time (*p* < 0.001) and significantly shorter LPA, MPA, and MVPA performance times than the standing worker group (*p* = 0.003, *p* = 0.001, and *p* = 0.004, respectively). However, regarding VPA, the performance time in the student group was significantly longer than those in the standing worker group and sitting worker group (*p* = 0.041 and *p* = 0.021, respectively) (Fig. 5B). The magnitude of the exercise (≥ 1.6 METs × hour) in locomotive activities was significantly lower in the sitting worker group than in the standing worker group (*p* = 0.047). Additionally, all categories of “Household” activities (METs (≥ 1.6) × hour, METs (≥ 3.0) × hour, and EE) of the student group were significantly lower than those of the standing worker group (*p* < 0.001, *p* < 0.001, and *p* < 0.001 respectively), and both categories of the magnitude of exercise of “Household” activities (METs (≥ 1.6) × hour and METs (≥ 3.0) × hour) were significantly lower in the student group than in the sitting worker group (*p* < 0.001 and *p* = 0.036, respectively). Similarly, all categories of “Household” activities were significantly lower in the sitting worker group than in the standing worker group (*p* = 0.004, *p* < 0.001, and *p* = 0.033, respectively) (Fig. 6A,B).

## Discussion

In the present study, the intensity of PAs (METs) and EE of participants were qualitatively and quantitatively evaluated using a WSD equipped with an accelerometer. The correlation between the performance time of each PA and step counts was weak, except for MPA and MVPA. Additionally, “Household” EE and step counts had a weak correlation. In the comparison of lifestyle, there was a significant difference in the mean performance time of each type of PA between the groups. There was also a significant difference in METs × hours and EE of "Household" activities between the standing worker and sitting worker groups.

Regarding the correlation of the step counts and performance time of each type of PA, the intensity of correlation depended on the type of PA. Many studies have been conducted to evaluate individual PA based on step counts^13,26–28^, but in the present study, it was clear that there were difficulties in estimating only the step counts in some of the intensities of PA due to the weak correlation. Thus, it is indispensable to quantitatively evaluate the PA for each intensity using a WSD. In addition, the correlation between the step counts and EE in “Household” activities was also weak. Furthermore, in the evaluation of the ratio of EE in “Locomotive” and “Household” activities, it was found that 79.8% of all participants expended most of their energy performing “Household” activities. Thus, “Household” activities are important for evaluating the PA of one’s daily life, and it is required to measure them by using a WSD.

Concerning the distribution of the step counts and weekly VPA performance time, 2.6% of participants were in the fourth quadrant, which means that those participants intentionally engaged in sports activities while walking less. In other words, by grouping participants in each quadrant using the WSD, it was possible to clarify the characteristics of each group, which cannot be seen by only conventional evaluation based on the average value of the entire group^12,18–20^. The evaluation of step counts is a simple and easy-to-use method for estimating daily physical activities. However, many people suffered physical inactivity in their lives and they try to perform sports activities including high-intensity activities to improve their situation, and those people increase these days. In those people, it is assumed that the step count is small while the VPA performing time is large. Thus, estimating the daily physical activities by step counts has a limitation for understanding the recent diversified lives. For solving the limitation of the step counts, using the WSD equipped with the accelerometer is more useful and that device may provide proper information to improve the physical inactivity daily lives.

In the comparison of lifestyles, students had low step counts and a long SB performance time, whereas their VPA performance time was long. However, workers had not only high step counts and a short SB performance time but also long LPA and MPA performance times. In the student group, many classes are held in the day in college and students spend most of the day sitting; therefore, it is assumed that this routine was the cause of the low step counts and long SB performance time. Long VPA performance time in the student group may have been caused by club activities and/or similar opportunities to perform sports activities^29^ In both worker groups (i.e., standing worker group and sitting worker group), workers probably move around in the workplace because of the working task they are performing^30,31^, and they do not have the opportunity to perform sports activities compared to students; hence, it is assumed that those situations are reflected in the WSD measurement data. From the above results, it was found in the WSD that the characteristics of the lifestyle in each group are reflected in both the step counts and performance time of each intensity of the PA. In addition, there was a significant difference in the PA performance time, “Household” METs × hour, and EE between the worker groups. The standing worker group performs a working task that requires changing places, carrying stuff, and so on^30,31^, and these working tasks may be distinguished from “Household” activities. In contrast, the sitting worker group mainly works at a desk^30,32^ and does not perform many intense activities. Detailed PA data obtained by the WSD clarified the difference between each group, and it is necessary to properly evaluate the PA situations in person.

The advantage of the WSD used in the present study was its ability to categorize the activity into "Locomotive" and "Household" activities and estimate the intensity of PA. Additionally, this device was reported to provide a good accurate estimation for the intensity of “Household” activities^21,22^, whereas other previous products had difficulty providing an accurate estimate^33^. Recently, with the acceleration of digitalization, work styles and lifestyles have diversified. Among them, the recent WSD can understand more detailed PA data than the step count measurement because of its comprehensive evaluation of the PA data. Additionally, it is not only able to perform an evaluation based on the average value of the entire group but it is also possible to evaluate what kind of activity an individual has performed. In particular, by distinguishing between "Locomotive" activities and "Household" activities and performing a PA evaluation, it is possible to characterize the lifestyle of individuals from the quantitative data. In general, "Household" activities are thought of as inactive, and if over half of one’s daily activities are accounted for by "Household" activities, it is recognized that he/she has an inactive lifestyle. However, the present results indicated that the EE was high in "Household" activities compared to the “Locomotive” activities in over half of the participants. These results suggest that “Household” activities burn energy to some degree and that they are important daily activities for many people. In previous studies, "Household" activities have not been sufficiently examined due to the limitations of measurement devices^33^. However, the “Household” activities may include the working task performed with sitting in the workers and those activities are may one of the reasons the “Household” EE was large in most of the participants in the present results. Thus, based on the present results, “Household” activities should be mentioned to evaluate individual activity situations. Those detailed data distinguishing between "Locomotive" activities and "Household" activities are important for not only health promotion research but also to a wide range of fields, such as rehabilitation and long-term care.

Recently, physical inactivity has become serious problem all over the world and many previous papers reported its association with non-communicable diseases^2–4^. Therefore, the proper management of daily physical activity is important for one's health care. The present result indicated that the WSD equipped with the accelerometer could obtain the detailed and quantitative physical activity situation reflect personal daily routine, and those data are useful for managing one's physical activity more than conventional methods such as questionnaires and pedometers.

The limitation of the present study is that the wearing time of the student group was significantly shorter than that of the other two groups, and it is necessary to perform an evaluation in which participants have the same wearing time in the future. Additionally, the present study reported an underestimation or overestimation of SB/LPA due to the lack of posture information in the WSD used in the present study^34^. Although sports activities are classified as "Locomotive" activities, some of the activities may generate similar acceleration as "Household" activities. Thus, it is suggested that the obtained data included both "Locomotive" activities and "Household" activities. In the future, it will be necessary to scrutinize the classification of "Locomotive" and "Household" activities during sports activities. In addition, there are few reports of classifying PAs into "Locomotive" activities and "Household" activities, and their recognition is extremely low. It will also be necessary to further examine the validity of the classification and apply it to large-scale research.

In conclusion, the accuracy of estimating the PA performance time based on step counts depends on the intensity of the PA, and detailed PA data can be obtained from the WSD. Additionally, “Household” activities are difficult to estimate based on step counts. These results indicate that the WSD measurement is more useful for evaluating the PA situation than the step count measurement. Moreover, the PA data measured by the WSD can be used to characterize the individual PA situation with respect to lifestyle. Especially, the important finding of the present study is that the differences in the PA between different occupations is reflected in "Household" activities.

## Methods

### Ethics statements

The present study design and protocol was approved by the Observation Research Ethics Review Committee of Osaka University Hospital (code: 19537). In the present study, informed consent was obtained from all subjects in writing and verbally. Additionally, prescribed information, such as research contents and inquiry, was described on the website of Orthopedic Surgery, Osaka University Graduate School of Medicine, and the subjects were given the opportunity to refuse. The present study followed the ethical recommendations for the study in humans, as suggested by the Declaration of Helsinki. Also, all methods were carried out in accordance with relevant guidelines and regulations.

### Participants and data collection

Data were collected from March 2019 to March 2021. Three hundred one subjects (105 men; age, 35.1 ± 14.4 years; BMI, 21.3 ± 2.7 kg/m^2^) participated in the present study. Participants included medical professionals working at hospitals in Osaka Prefecture, Japan, college students, graduate students, faculty members (Graduate School of Medicine), researchers, and personnel (office clerks and shop clerks). The physical data (height and weight) of the participants were collected, and the BMI was calculated as weight (kg) divided by squared height (m^2^).

To determine the relationship between PA and lifestyle, participants were divided into student, standing worker, and sitting worker groups. The distinction in the present study was referred to previous reports. In those reports, subjects responded to questionnaires about their occupational activity patterns such as desk works requiring constant sitting or standing works requiring moving around, and categorized based on the results^30–32^. Most sales workers answered their jobs are often standing. On the other hand, Clerical and administrative workers and Professionals answered their jobs are often sitting^31^. In the present study, medical worker and store staff were defined as standing workers, representing standing jobs^30,31^ and office clerk, faculty members, and researcher were defined as sitting workers, representing sitting jobs^30,32^.

### Measurement of physical activity using a wearable sensor device

Daily PAs of the participants were measured using a WSD equipped with a tri-axial accelerometer (Active Style Pro, HJA-750C, Omron Healthcare, Kyoto, Japan). The range of the acceleration data of each axis was ± 6 G, and the resolution was 3 mG in the device. Additionally, the device recognizes the activities into “Locomotive” or “Household” based on the resultant acceleration calculated from the ratio determined based on high-pass filtered (performed at rate of 0.7 Hz) data and the raw data. Then, from the resultant acceleration calculated at 10-s epochs, “Locomotive” and “Household” activities are discriminated, and the METs of each activity are estimated^21,22^. For the measured acceleration of physical activity, the filtered acceleration is ACC_fil_, the acceleration before processing is ACC_unfil_, and when the ratio of ACC_unfil_: ACC_fil_ is ≥ 1.16, it is distinguished from "Household" activity, and it is 1.3435 + 0.0196 × ACC_fil_. METs are calculated. On the other hand, when the ratio of ACC_unfil_: ACC_fil_ is < 1.16, it is a "Locomotive" activity, and METs are calculated by 1.1128 + 0.0086 × ACC_fil_^21,22^. The participants were instructed to wear the accelerometer around their waist for 10 h or more each day (excluding sleeping and bathing) for 7 consecutive days^13,35^. Participants who collected the accepted daily data for 4 days or more were included in the data analysis^13,35^. In addition, if there was a loss or failure, including submersion during wearing, the participant was excluded from the analysis^13^. The data of METs measured by 10-s epochs, step count, EEs obtained by the accelerometer were extracted using the manufacturer’s original application (activity meter application, version 2.0, Omron Healthcare, Kyoto, Japan). Based on the data, the average number of steps per day, SB, PA, and EE were calculated. The average time of VPA performed during the day and week was also calculated. Furthermore, the product of weekly activity intensity and time (METs × hours) was calculated. For comparison with the pedometer, scatter plots were created with step counts, SB, each type of PA, and "Locomotive" and "Household" EE. In addition, in order to evaluate the lifestyle of each participant, a scatter plot was created with two items, the step counts per day and the performance time of VPA per week, from the health outcomes^25^ and the PA guidelines established by each country and institution^7^. Additionally, the average values of the step counts and performance times of SB and each type of PA in the three groups (student, standing worker, sitting worker) were compared.

### Data analysis

The physical data of the subjects and the data obtained by the accelerometer are shown as the mean ± standard deviation. For comparison of the three groups, the Steel–Dwass test was used to analyze all terms. In addition, the Pearson correlation coefficient test was used to examine the correlation between SB/PA/EE and step counts. Statistical analyses were performed using the statistical calculation software (R version 4.0.2, R Foundation for Statistical Computing, Vienna, Austria), and *p*-values < 0.05 were considered statistically significant.
